# Evaluation of different bermudagrass germplasm at physiological and molecular level under shade along longitudinal and latitudinal gradients

**DOI:** 10.1186/s12870-024-05384-y

**Published:** 2024-07-15

**Authors:** Maryam Noor, Muhammad Kaleem, Muhammad Tanveer Akhtar, Guilan Feng, Jingxue Zhang, Usman Nazir, Jibiao Fan, Xuebing Yan

**Affiliations:** 1 College of Animal Science and Technology, Yangzhou University, Yangzhou, 225009 China; 2 Department of Botany, University of Agriculture, Faisalabad, 38040 Pakistan; 3 College of Horticulture and Landscape Architecture, Yangzhou University, Yangzhou, 225009 China

**Keywords:** *Cynodon dactylon*, Shade, Morpho-physiology, Gene expression, Longitude, Latitude

## Abstract

Responses of turfgrass to shade vary in individual species, and the degree and quality of low light; therefore, the selection of low light tolerant cultivars of turfgrass is important and beneficial for turf management rather than other practices. The stolons of thirteen bermudagrass genotypes were planted with two treatments and three replications of each treatment to establish for one month in the Yangzhou University Jiangsu China greenhouse. The established plants were transferred outside of the greenhouse, and 50% shading was applied to them with a black net. After 30 days of stress treatment, the morpho-physiological and biochemical analyses were performed. The expression of genes such as *HEMA, HY5, PIF4*, and *Cu/ZnSOD* was assessed. *Cynodon dactylon* is a C_4,_ and perennial that grows as lawn grass and is used as forage. Based on different indicator measurements, the most shade-tolerant germplasm was L01 and L06 along the longitudes and L09 and L10 along the latitudes. At the same time, L02 and L08 were more susceptible, respectively. However, germplasm showed greater tolerance in higher latitudes while longitudinal plants showed less stress response. The current study aimed (1) to screen out the most shade-tolerant *Cynodon dactylon* genotype among 13 along longitudinal and latitudinal gradients in China. (2) to examine morpho-physiological indicators of different bermudagrassgenotypes; (3) to evaluate if and how differences in various indicators of bermudagrass correlated with geographic region. This study will significantly advance the use of *Cynodon* germplasm in breeding, genomics, management, nomenclature, and phylogeographical study. It will decisively define whether natural selection and migration can drive evolutionary responses for populations to adapt to their new environments effectively.

## Introduction

Light, being a signal, is an indispensable environmental factor influencing turfgrass growth through plant morphogenesis and photosynthesis. It can induce, regulate, and promote plant growth and cell differentiation and enhance development [47]. Grasses are highly sensitive to light changes. Reduction in light intensity normally combines with several other significant environmental factors, such as change in light quality, reduced airflow, tree-root competition, moderate temperatures, increased leaf wetness and relative humidity [48]. Regarding morpho-physiological characteristics, turfgrasses have developed specific countermeasures to adapt to capture and absorb more light energy by changing light intensity and quality [35]. The most significant changes in red/far-red (R/FR) ratios under shade [8]. Plants have two photosensitive pigments, Pfr (active) and Pr (passive), responsive to external light stimulation. Phytochromes with various types are red/far red-light sensors that act as photoreceptors to transmit plant light signals [33]. Plants contain PHYA, PHYB, PHYC, PHYD, and PHYE, which are light-sensitive proteins that absorb and convert red into far-red light and play a critical role in regulating plant growth and development. PHYA is involved in seed germination, while PHYB is involved stem elongation and flowering. PHYC, PHYD, and PHYE are involve in the circadian rhythm regulation of plants [36].

As fundamental ecological obstacles, turfgrasses can beautify and protect the earth, ecology and microclimate. It provides visitors with refreshment places and reduces desert islands' hot climatic effects [18]. Taxonomically, bermudagrass (*Cynodon dactylon* L.) belongs to the family *Poaceae* and the tribe *Cynodonteae* and grows extensively in warmer months of the year so, called warm-season and C_4_ turfgrass [15]. Many studies have been done on it because of its extensive use as lawn grass, pasture, forage, and soil stabiliser. *C. dactylon* has outstanding growth habits such as lush green colour, aggressive growth and high turf density. Additionally, it is a tolerant of several severe environments and soil types, such as drought, heat, and wear, but it is susceptible to shaded conditions. Normally, it has a high light compensation point, requires 8 h of light, and a luminosity level between 15,000 and 16,000 lx per day. Therefore, they are highly sensitive to shade stress [55]. Bermudagrass ranked least shade tolerant compared to other warm-season grasses [9]. High buildings, tall trees, bushes, and other physical and natural objects provide high buildings, tall trees, bushes, and other physical and natural objects provide shady areas, which is challenging for grass managers [54]. With the increasing number of high buildings in urban areas, tall trees and other natural objects in landscaping result in shade, which changes the light intensity and light hours of the lawn. In this challenging environment, grass grows at the bottom of a wild community [50]. It is estimated that 20% to 25% of the grass is under shade in the U.S.A., while 50% of the total grassland in China is subjected to shade. Thus, shade is one of the worst sources of turf deterioration [52].

To survive in shady areas, morphologically *C. dactylon* exhibits elongated stolon, narrow leaf blades with reduced leaf area, longer internodes with reduced internode diameter and the number of tillers, but this results in a gradual reduction in turf quality and ground coverage, in addition, it experiences to other diseases, pests attack and recession [26].

Physiologically, shade can affect the primary reactions of photosynthesis, decreased chlorophyll content, high osmotic potential and reactive oxygen species (ROS), electron transfer, photosynthetic phosphorylation and net carbon assimilation and ultimately results in depressed photosynthetic efficiency [28]. Excessive ROS results in cell membrane damage and lipid peroxidation; a clear demonstration occurred that ROS is involved in the expression of several genes and signal transduction pathways [2]. To cope with high ROS content, the plant activates several enzymatic antioxidants such as SOD, POD and CAT. Under shade, superoxide dismutase is a basic enzyme for maintaining regular physiological processes and managing oxidative damage by quickly converting O_2_^−^ to O_2_ and H_2_O_2_ [27]. Therefore, to investigate the expression level of responsible genes for encoding the following enzymes was necessary. There are several genes encoding SODs, each of which produces an isozyme.

Additionally, these SOD isoenzymes are specifically localized in different organelles such as chloroplasts, mitochondria, peroxisomes, and cytosol [46]. *Cu/ZnSOD* is a gene in both the chloroplasts and the cytosol of higher eukaryotes, including bermudagrass [22]. In our study, some ROS scavenging gene’s *CuZn/SOD* (copper-zinc superoxide dismutase) [12], upregulated in bermudagrass in mitochondria [41].

In photosynthesis, the chlorophyll content and photochemical efficiency increased under shade by activating the phytochrome interacting factor gene as *PIF4*. It is closely related to shade avoidance mechanisms. It affects plant morphogenesis by directly interacting with phytochrome molecular factors [32], photosynthetic performance and optical signalling cascade improved by up-regulating *HEMA* (glutamyl t-RNA reductase family) and *HY5* (Basic-leucine zipper (bZIP) family) genes [23].

China is rich in bermudagrass germplasm resources with vast territory zones across large longitude, latitude, and altitude ranges [56]. Geographic or environmental gradients profoundly affect the adaptive selection of a genotype, which is observed in its molecular and phenotypic traits. Geographic location or elevation directly influences factors such as precipitation, temperature, nutrient availability, photoperiod, growing season length, and other biotic agents [13]. Previous studies have shown that geographical locations significantly affect morphological variations [16]. Morphological variation in response to environmental changes at different geographic positions is common in *C. dactylon* [19]. The pronounced east-to-west longitudinal variations in morphological traits in bermudagrass show that longitudes plays a major role in morphological variation. We observed that morphological trait sizes tended to increase with decreasing longitude for the plants to adapt to the changing environment [50]. A study of 260 accessions of bermudagrass germplasm indicated extensive morphological variation in *C. dactylon* populations along longitudinal gradients, which has been found in some other studies [34]. The longitude gradients showed null or reduced stress response because suitable annual rainfall, precipitation and temperature do not significantly affect bermudagrass plant growth under stressed conditions [6]. The relationships between latitude and phenotypic variation (particularly phenology traits such as bud set) have been established previously in other species [7]. *Cynodon* morphological variation along environmental gradients demonstrated that their geographic patterns were shaped by environmental factors (climatic and edaphic gradients) and phylogenetic differences. Such weather conditions contributed positively to rice yield by increasing the number of panicles per hill and the number of spikelets per panicle significantly [1].

According to the results, a significant relationship was observed between the latitude and several bermudagrass morphological characteristics, indicating that latitude was the key factor influencing the evolution of *Cynodon* [31]. Meanwhile, for the latitudinal region, it was observed that the germplasm in higher latitudes depicts higher tolerance because of poor environmental conditions such as low precipitation, insufficient water, sunlight and low temperature, as the higher the latitude, the smaller the effect of stress will be. However, the genetic reason behind shade plasticity across the longitudinal and latitudinal gradient is currently obscure. Several previous studies have established that light sensitivity strongly correlates with the latitudinal site of origin, suggesting that genetic adaptation to latitudinal gradients may be responsible for differences in light responsiveness [20].

## Methodology

### Plant material

The material was collected from 13 sites by taking Zhengzhou as the central point with a similar longitude of 34°54' N and latitude 113°38' E, 7 from a longitude between 105°57' E to 119°27' E with a similar latitude while 6 from a latitude between 22°35' N to 36°18' N with similar longitude. All the collected material was grown in the experimental field of Yangzhou University, Yangzijin Campus, Jiangsu Province, China (32°31′05″N, 119°24′03″E). Total annual precipitation and mean annual temperature were provided by the China Meteorological Administration of the collection locations of the plants (Table 1).

**Table 1:** *Cynodon dactylon* populations collected from different longitudes and longitudes in China

Gradient	Population code	Localities	Latitude(N)	Longitude(E)	Altitude/m	AAT(°C)	AAP(mm)	Habitat	Sunshine(H/Y)
Longitude gradients	L01	Tianshui	34°32′43"	105°57′34"	1050	11.4	500.7	Roadside	2132.405745
	L02	Baoji	34°21′54"	107°41′03"	630	13.5	645.9	Ditch side	1983.372335
	L03	Sanmenxia	34°42′29"	111°03′49"	340	14	558.1	Roadside	2133.710142
	L04	Zhengzhou	34°54′04"	113°38′20"	90	14.7	640.8	Roadside	2128.736402
	L05	Shanxian	34°46′31"	116°09′11"	30	14.2	621.4	Roadside	2226.170563
	L06	Zaozhuang	34°38′48"	117°49′20"	89	14.4	820.3	Roadside	2285.693077
	L07	Lianyungang	34°46′09"	119°27′06"	50	14.5	883.9	Island	2302.408824
Latitude gradients	L08	Zhongshan	22°35′40"	113°23′17"	0	22.0	1846.8	Roadside	1811.973975
	L09	Youxian	27°00′59"	113°23′07"	342	18.1	1518.4	Roadside	1535.434412
	L10	Linxiang	29°28′32"	113°26′48"	45	16.8	1582.5	Roadside	1788.263999
	L11	Xiaochang	31°18′59"	114°02′15"	30	16.8	1138.0	Roadside	1946.221665
	L12	Zhumadian	33°09′47"	114°03′45"	85	15.2	990.4	Arable Land	1959.350685
	L13	Cixian	36°18′40"	114°11′51"	107	13.4	509.2	Roadside	2265.703006

*AAT(°C)* Annual average temperature/°C, *AAP(mm)* Annual average precipitation/mm; Sunshine duration(hours/year)

### Experimental design and shade treatment

In the following experiment stolons of 13 bermudagrass germplasm with two treatments (Control and Shade) and three replications of each treatment with complete randomized block design were established in plastic pots (diameter 16 cm, height 17.5 cm) filled with clay loam soil and yellow sand with a ratio of 1:1 in the greenhouse of Wenhui road campus of Yangzhou University Jiangsu China at 25 °C with a 12/12 h photoperiod for one month to establish the plant. The established plants were transferred outside of the greenhouse for shading treatment. The shading structure (cube size: 3 × 3 × 2.5 m) was built from PVC and covered with polyethylene netting (Jurong Huanan Plastic Products Co. Ltd., China) for 50% shading (luminosity level between 9000 and 8000 lx). Non-shading plants served as the control. After the application of one month of shade stress, the morphological parameters were examined, and plant leaves were stored for further analysis.

### Statistical analysis and design

The current experiment was planned as a completely randomized design with two treatments and three replications to screen out the shade tolerance in *C.dactylon* genotypes. For morphological data interpretation, the mean ± SE values were curated by Tukey’s HSD test, by using the “agricolae” package of R software (Mendiburu, 2020). For physio-biochemical indicators, R statistical software validated the statistical interpretations (R Core Team, 2021) in an R-integrated environment (R Studio Team, 2021). The replicate values are subjected to a two-way analysis of variance ANOVA at *p* ≤ 0.05 to evaluate the difference between varieties under control and shade applications. The ellipsed PCA was done by using the package “FactoMineR and ggplot2” by R software. The ggbiplot2 package computed the correlation matrix. The clustered heatmap was made by using the R customized code “heatmap”, and the hierarchical dendrogram was constructed by the "factoextra&quot customized package. The Graph-Pad Prism8 (Graph Pad Software Inc., San Diego, CA) was used to evaluate the overall changes in gene expression of bermudagrass.

### Morphology

The morphology of each bermudagrass germplasm was examined thoroughly, including plant height, stolon length, internode length, internode diameter, leaf length, leaf width and number of tillers. Plant height (cm) was measured with measuring tape. Stolon length (mm), internode length (mm) and diameter (mm) were measured with vernier caliper by selecting the random stolon from the whole pot. For leaf length and width (mm), the fully expanded third leaf from the apical meristem was selected, measured with a vernier calliper, and averaged by four replicates. Additionally, the number of tillers was counted quantitatively according to the method [23]. On the last day of the experiment, the fresh weight was obtained, and for the dry weight, the plants were oven-dried at 80 °C for 8 h till the constant weight.

### Relative water content

To assess relative water content (RWC), the method of Lu et al., [25] was followed. The fully expended fourth leaf was detached and placed on the weighing balance inside the same greenhouse. FW (fresh weight) of the detached leaves were quantified every 1-h intervals for up to 8 h, then immersed in water for 3–4 h until the weight of leaves was constant. The turgid weight as (TW) was measured. The dry weight (DW) was determined after 16 h incubation at 80 °C. The RWC were calculated by using the following formula:

$$RWC\,(\%) = (FW-DW)/(TW-DW) \times\,100$$

### Electrolyte leakage (EL)

For the EL assay [42] method was applied, in short, 0.1 g leaves were rinsed with distilled water and immersed in 10 ml of double deionized water. The mixture was then shaken at room temperature for 6 h to ensure the sample and water were mixed thoroughly. The sample's initial conductivity (Ci) was measured using a conductivity meter (Leici-DDS-307A, Shanghai, China). The sample was then boiled for 20 min and left to cool to room temperature. The conductivity of the sample was then measured again to determine the maximum conductivity (Cmax).

$$Relative\,EL (\%) = (Ci/Max)\,\times\,100$$

### Chlorophyll contents

Plant leaf chlorophyll was determined by the method of Fan et al., [11] with slight modifications. Briefly, 0.1 g of fresh leaf samples was homogenized into 1 mL of 80% acetone that contained grinding beads in 1.5 mL centrifuge tubes. Then, the tubes were kept in a grinding machine for 3 min and centrifuged at 15,000 rmp for 5 min at room temperature. The absorbance of the extract was observed at 647 nm and 664 nm with a spectrophotometer. Chlorophyll and carotenoid content was calculated with the following formula.

$$Chl-\,content\,(*mg.\,L^{-1})\,=\,20.2\times\,OD647+8.02\times\,OD664$$

### Photosynthetic traits

Net CO_2_ assimilation rate (*A*), transpiration rate (*E*) and stomatal conductance (*gs*) of fully expanded matured leaf were estimated using a Li Core 6400 portable photosynthesis system (Li-Core Inc, USA) according to Cao et al., [3] method. Photosynthetic traits were assessed in the early morning (9:00–11:00 AM) under moderate light conditions, PAR set at 750 μ mol^−1^ s^−1^ and CO_2_ at 350 μ mol^−1^ s^−1^.

$$WUE\,=\,A/E\,A\,represents\,the\,net\,assimilation\,and\,E\,transpiration\,rates$$

### Crude enzyme extraction

A 0.1 g sample of fresh leaf was ground with liquid nitrogen and ground into a fine powder. The 4 ml of sodium phosphate buffer with 150 mM and pH 7.0, was pre-cooled at 4 °C, and mixed in the powder. Then, the mixture was shifted into a 10 ml tube and centrifuged at 12000rmp and 4 °C for 20 min. The supernatant obtained at the end was the crude enzyme to be determined.

#### Malondialdehyde contents

The lipid peroxidation was determined by measuring the amount of MDA as previously described by the method Shi et al., [43], with the thiobarbituric acid (TBA). A total of 1 mL crude enzyme solution was added into 2 mL MDA reaction buffer that includes 0.6% (v/v) thiobarbituric acid (TBA) and 10% (v/v) trichloroacetic acid (TCA). The reaction solution was heated at 95 °C for 30 min in a water bath, cooled at room temperature, and centrifuged at 12,000 rpm at 25 °C for 10 min. The supernatant was obtained for absorbance at 450 nm, 532 nm and 600 nm. MDA content was calculated by the following formula: (645 × (OD_532_-OD_600_)—0.56OD_450_) × 0.015/ W.

#### H_2_O_2_ level

0.1 g fresh leaves were mixed with liquid nitrogen, grounded, and then uniformly mixed with extraction buffer (50 mM sodium phosphate buffer, pH 7.8). Then, centrifuged at 12,000 rpm for 30 min at 4 °C, 1 ml of supernatant was homogenized completely with 1 ml of 0.1% titanium sulphate in 20% H_2_SO_4_ (v/v) for 10 min. After centrifugation at 12,000 rpm for 10 min at room temperature, the absorbance of the mixture was measured at 410 nm with known H_2_O_2_ concentration as standard by following the Shi et al., [44] procedure.

#### Proline contents

To determine proline content Fan et al., [11] method was followed as 0.2 g fresh leaves were cut into small pieces, added 5 ml of sulfosalicylic acid, and then kept the tubes in a water bath at 98 °C for 10 min. After cooling at room temperature, 2 ml of the following plant sample was mixed with 3 ml of mixture solution of 2.5% ninhydrin mixed in glacial acetic acid and phosphoric acid in 3:2 and 2 ml of acetic acid then kept in the water bath at 98 °C for 40 min, after cooling at room temperature 5 ml of methylbenzene and shanked for 30 min to obtain supernatant. Finally, the absorbance was measured at 520 nm with a spectrophotometer.

#### Enzymatic antioxidants

To determine the SOD activity Shi et al., [44] method was followed, 0.005 mL crude enzyme was extracted, was mixed with 3 mL reaction mixture, which includes 2.2 ml sodium phosphate buffer (50 mM, pH 7.8), 0.4849 gmethionine, 0.0186 gethylene diaminetetraacetic acid (EDTA), 0.0038 griboflavin, 0.0153 g nitro blue tetrazolium (NBT) and 0.003 ml reaction solution without crude enzyme was set as standard. The mixture was irradiated under a 4000 lx fluorescent lamp for one hour for the chromogenic reaction solution. The absorbance was calculated at 560 nm using a spectrophotometer. One-unit SOD activity was mentioned as the amount of SOD required inhibiting NBT reduction by 50%.

The POD activity was determined by the following Shi et al., [43]. The 40 μL of the crude enzyme was mixed into a 3 mL reaction mixture, which includes sodium acetate acetic acid buffer (pH 6.0), 0.037 mL guaiacol (guaiacol was dissolved in 50% ethanol solution) and 0.056 mL 30% H_2_O_2_. The absorbance of the mixture at 460 nm was increased per minute and recorded for 3 min.

#### Gene expression

The total RNA of bermudagrass was executed using a Freezol Reagent kit (Nanjing Novizan Co., Ltd. China). RNA quality was determined using a NanoDrop ND-1000 spectrophotometer (NanoDrop Technologies, Rockland, DE, USA) and Bioanalyzer 2100 system (Agilent Technologies, CA, USA) was used to test the quantity and integrity of RNA samples. The first-strand complementary DNA (cDNA) was obtained with the kit (Nanjing Novizan Co., Ltd. China) according to the manufacturer’s instructions. The qRT-PCR was carried out via an ABI System of Fast Start Universal SYBR®Green Master Mix (Roche) using kit (Nanjing Novizan Co., Ltd. China). The primers used for qRT-PCR are listed in table (Table 2). *CdACTIN* was used as a reference gene (Chen, [6]). Each sample had three biological replicates [40].

**Table 2:** Primers used in qRT-PCR

**GENE**		**Primers (5**'**-3**'**)**
*HEMA*	F	ACCTGCATCTGAGCTTAGGG
	R	TCTGTCCTTCACCCAGAACC
*PIF4*	F	AAGCTTCTACCAGCAGCAGA
	R	TGTGCTGTCAGAAGGAAACG
*HY5*	F	GTTGCTGAGGAACCGTGTG
	R	CAGCTCGGAGTTCCTCTTCT
*Cu Zn /SOD*	F	TCTTCCACCAGCATTTCC
	R	AGGCGTGGCTGAGACAAC
*CdActin* *(Control)*	F	AGGCATCCAACCAGCAGAGA
	R	ACTCAGCACATTCCAGCAGAT

## Results

### Morphology

Plant height and internode diameter decreased significantly under shade stress along longitudinal and latitudinal gradients. Under control treatment, plant height was highest in L02 and L07 and minimum in L04 in control and shade as well. Thicker internode diameter was observed in L02 and L06, but L05 showed the least diameter thickness under control. When shade stress was present, the least reduction in diameter was observed in L01 and L05 (Table 3). An effective increase in stolon, internode, leaf length and leaf width was observed when subjected to shade stress (Table 3). Stolon length increased in L01 and L06 under normal conditions although under shade stress L01 and L06 maintained the highest stolon length compared to other locations. The longest internodes were observed in L04, L05 and the smallest in L07 under controlled condition. So, the same trend was maintained under stressed conditions. Under normal conditions, the longest leaves were observed in L04 and L07 and shortest in L02. When shade was applied, L06 and L04 revealed increased LL, while L02 showed the least leaf length. Leaf width increased to some extent under a stressed environment but did not change significantly in control conditions. However, L01 showed maximum, and L02 showed minimum LW under shade (Table 3).

**Table 3:** Morphological traits of *Cynodon dactylon* under control and shade stress along longitude gradient

Var	PH	SL	IL	ID	LL	LW
	Control					
L01	32.00 ± 1.00^cA^	16.11 ± 1.79^aB^	25.21 ± 1.15^cB^	0.63 ± 0.00^bA^	51.72 ± 0.98^ dB^	2.14 ± 0.01^aB^
L02	39.00 ± 1.00^aA^	15.67 ± 0.69^aB^	29.47 ± 0.99^bA^	0.88 ± 0.01^aA^	47.46 ± 1.07^eB^	1.08 ± 0.01^bA^
L03	31.33 ± 1.33^cA^	10.11 ± 0.68^bB^	35.19 ± 0.15^aB^	0.75 ± 0.01^bA^	55.66 ± 1.10^cB^	2.23 ± 0.01^aB^
L04	28.00 ± 0.58^cA^	14.56 ± 1.09^aB^	38.07 ± 0.77^aB^	0.68 ± 0.01^bA^	74.02 ± 1.66^aB^	2.68 ± 0.01^aB^
L05	36.67 ± 0.88^bA^	15.00 ± 0.38^aA^	36.11 ± 1.16^aA^	0.57 ± 0.01^cA^	43.00 ± 1.47^fB^	2.15 ± 0.01^aA^
L06	29.67 ± 0.88^cA^	16.67 ± 0.69^aB^	31.93 ± 1.00^bB^	0.83 ± 0.01^aA^	71.14 ± 0.61^bB^	2.15 ± 0.01^aB^
L07	40.33 ± 0.67^aA^	9.11 ± 0.11^bB^	23.51 ± 0.93^cB^	0.91 ± 0.01^aA^	75.33 ± 1.62^aB^	2.52 ± 0.00^aB^
	Shade					
L01	31.33 ± 0.33^aA^	22.11 ± 0.40^aA^	33.70 ± 0.67^bA^	0.61 ± 0.00^aA^	86.91 ± 1.37^aA^	3.02 ± 0.01^aA^
L02	31.00 ± 0.58^aB^	17.33 ± 0.96^bA^	30.43 ± 0.51^bA^	0.73 ± 0.00^aB^	58.39 ± 0.29^cA^	1.37 ± 0.01^bA^
L03	23.67 ± 0.88^bB^	12.33 ± 1.20^cA^	38.54 ± 0.84^aA^	0.56 ± 0.01^bB^	68.86 ± 1.09^bA^	2.48 ± 0.01^aA^
L04	21.00 ± 0.58^bB^	18.78 ± 0.73^aA^	41.14 ± 0.85^aA^	0.61 ± 0.01^aA^	87.21 ± 1.13^aA^	2.84 ± 0.01^aA^
L05	32.67 ± 0.88^aB^	17.89 ± 0.87^bA^	38.52 ± 0.81^aA^	0.52 ± 0.01^bA^	57.95 ± 0.30^cA^	2.33 ± 0.01^aA^
L06	22.33 ± 0.88^bB^	19.90 ± 1.29^aA^	34.82 ± 1.01^bA^	0.65 ± 0.00^aB^	88.55 ± 0.40^aA^	2.36 ± 0.01^aA^
L07	33.67 ± 0.88^aB^	11.33 ± 0.33^cA^	26.57 ± 0.32^cA^	0.62 ± 0.01^aB^	86.95 ± 0.68^aA^	2.66 ± 0.02^aA^

Means are provided with error bars. Small letters indicate a significant (*p* ≤ 0.05) difference between locations and capital letters between treatments of *Cynodon dactylon*. Abbreviations are given at the end of the manuscript

*Abbreviations*: *PH* plant height, *SL* stolon length, *IL* internode length, *ID* internode diameter, *LL* Leaf length, *LW* leaf width

Whereas, along the latitudinal gradient, plant height was maximum in L09 and L11 under control but L09, L10 showed maximum height. Meanwhile, minimum height was observed in L08 under both (normal and shade) conditions. Stolon length was higher in L10 and L11 and decreased in L08 under control, when subjected to shade L10 and L13 maintained higher SL but significantly decreased in L08. Internode and leaf length were enhanced more positively in L12 and L13, but L09 showed the least IL in a normal environment. In stressed condition more LL was shown by L08 and L10 under control, and the same trend was followed under stressed environments. The internode diameter was maximum in L11, under normal, and minimum in L13. When subjected to shade, L10 showed maximum diameter and minimum of L08 and L09. Leaf width did not change more significantly under normal conditions among all locations, but under shade, L10 showed more LW (Table 4).

**Table 4:** Morphological parameters of bermudagrass under controlled and shade stress along latitudinal gradient

Var	PH	SL	IL	ID	LL	LW
	Control					
L08	32.33 ± 1.45^dA^	10.44 ± 0.11^cA^	27.11 ± 1.25^cA^	0.72 ± 0.01^aA^	67.28 ± 0.93^bB^	1.75 ± 0.00^bA^
L09	47.00 ± 1.53^aA^	16.22 ± 0.78^bA^	26.46 ± 1.11^ dB^	0.72 ± 0.00^aA^	63.90 ± 1.52^cB^	2.05 ± 0.01^aA^
L10	41.33 ± 0.67^bA^	18.89 ± 0.73^aB^	26.84 ± 0.47^ dB^	0.82 ± 0.01^aA^	47.49 ± 0.98^fB^	2.43 ± 0.00^aB^
L11	47.00 ± 1.00^aA^	18.67 ± 0.51^aA^	36.24 ± 1.18^bB^	0.91 ± 0.01^aA^	58.05 ± 1.03^ dB^	1.55 ± 0.02^bA^
L12	42.00 ± 1.15^bA^	20.11 ± 0.99^aB^	44.59 ± 0.80^aB^	0.86 ± 0.01^aA^	86.49 ± 1.82^aB^	2.16 ± 0.01^aA^
L13	39.00 ± 0.58^cA^	19.89 ± 1.39^aB^	43.13 ± 1.54^aB^	0.67 ± 0.02^aA^	85.54 ± 1.21^aB^	2.44 ± 0.02^aA^
	Shade					
L08	23.67 ± 0.88^cB^	11.97 ± 0.35^cA^	29.97 ± 0.86^dA^	0.53 ± 0.01^ dB^	77.63 ± 1.15^cA^	1.97 ± 0.01^cA^
L09	39.33 ± 0.33^aB^	18.52 ± 0.35^bB^	29.31 ± 0.53^dA^	0.61 ± 0.01^cB^	83.71 ± 0.74^bA^	2.22 ± 0.01^bA^
L10	38.67 ± 0.33^aA^	25.08 ± 0.37^aA^	33.17 ± 0.75^cA^	0.78 ± 0.00^aB^	96.07 ± 0.76^aA^	3.06 ± 0.00^aA^
L11	29.00 ± 0.58^bB^	19.67 ± 0.38^bA^	39.03 ± 1.05^bA^	0.73 ± 0.01^bB^	67.95 ± 0.55^dA^	1.77 ± 0.02^cA^
L12	31.00 ± 0.58^bB^	20.56 ± 1.31^bA^	47.26 ± 0.33^aA^	0.73 ± 0.01^bB^	97.59 ± 1.62^aA^	2.45 ± 0.01^bA^
L13	36.67 ± 1.20^aA^	22.06 ± 1.15^aA^	47.19 ± 1.03^aA^	0.40 ± 0.01^eB^	97.37 ± 0.89^aA^	2.63 ± 0.01^bA^

Means are provided with error bars. Small letters indicate a significant (*p* ≤ 0.05) difference between locations and capital letters between treatments of *Cynodon dactylon*. Abbreviations are given at the end of the manuscript

*Abbreviations: PH* plant height, *SL* stolon length, *IL* internode length, *ID* internode diameter, *LL* leaf length, *LW* leaf width

### Growth

The fresh and dry weight of plant were measured in the growth net tiller, electrolyte leakage. NT, FW and DW decreased while, EL increased significantly under shade stress. EL was more in L04 in control and L06 under shade, but L01 showed minimum EL under both (normal and stress) conditions. Higher tillers were counted in L07 and minimum in L02 under normal. The higher number of tillers maintained by L05 and L03 showed the least NT under shade, L11 and least in L02 were normal, and L01 and L03 and L05 were. More FW and DW were calculated of L05 and the least of L02 under normal and shade conditions (Table 5).

**Table 5:** Growth parameters of *Cynodon dactylon* under controlled and shade stress along longitude

Var	NT		EL		FW		DW	
	Control	Shade	Control	Shade	Control	Shade	Control	Shade
L01	19.00 ± 0.58^bA^	18.00 ± 0.58^aA^	6.58 ± 0.26^eB^	10.19 ± 0.38^dA^	16.51 ± 0.06^cA^	14.84 ± 0.03^aB^	10.96 ± 0.03^aA^	9.93 ± 0.04^aA^
L02	18.33 ± 0.67^bA^	14.33 ± 0.67^bB^	14.82 ± 0.38^cB^	21.66 ± 0.18^bA^	7.58 ± 0.02^eA^	3.51 ± 0.01^ dB^	4.55 ± 0.04^cA^	2.35 ± 0.02^cB^
L03	20.00 ± 1.00^aA^	11.67 ± 0.88^cB^	10.55 ± 0.07^ dB^	14.77 ± 0.69^cA^	14.15 ± 0.03^cA^	7.24 ± 0.02^cB^	8.26 ± 0.04^bA^	3.69 ± 0.01^bB^
L04	20.00 ± 1.15^aA^	13.67 ± 0.67^bB^	23.21 ± 1.28^aB^	29.96 ± 0.35^aA^	17.67 ± 0.02^bA^	12.67 ± 0.03^bB^	9.97 ± 0.04^aA^	3.73 ± 0.02^bB^
L05	21.00 ± 0.58^aA^	20.00 ± 0.58^aA^	15.91 ± 0.04^bB^	20.62 ± 0.07^bA^	23.34 ± 0.02^aA^	16.50 ± 0.05^aB^	12.85 ± 0.05^aA^	7.79 ± 0.01^aB^
L06	19.67 ± 0.67^bA^	12.67 ± 0.33^cB^	16.12 ± 0.06^bB^	31.29 ± 0.41^aA^	11.45 ± 0.03^dA^	5.39 ± 0.01^cB^	6.97 ± 0.03^bA^	2.88 ± 0.01^cB^
L07	23.67 ± 0.88^aA^	16.33 ± 0.88^bB^	15.87 ± 0.08^bB^	23.15 ± 0.72^bA^	19.01 ± 0.02^bA^	11.67 ± 0.02^bB^	10.96 ± 0.11^aA^	4.73 ± 0.03^bB^

Means are provided with error bars. Small letters indicate a significant (*p* ≤ 0.05) difference between locations and capital letters between treatments of *Cynodon dactylon*. Abbreviations are given at the end of the manuscript

*Abbreviations: NT* net tiller, *EL* electrolyte leakage, *FW* fresh weight, *DW* dry weight

Under the latitudinal gradient, the net tiller was higher in number in L11 and lowest in L08 under normal. When shade was applied, the L10 showed higher, and L08 showed lower NT.. More FW, DW and a lower rate of EL were observed in L10, while reduced FW, DW and a higher rate of EL in L12 under both (control and shade) treatments (Table 6).

**Table 6:** Growth traits of bermudagrass under normal and shaded conditions along latitudinal gradient

Var	NT		EL		FW		DW	
	Control	Shade	Control	Shade	Control	Shade	Control	Shade
L08	17.33 ± 0.67^cA^	11.67 ± 0.67^cB^	14.56 ± 0.48^bB^	21.99 ± 0.32^bA^	24.51 ± 0.02	16.50 ± 0.00^bB^	15.32 ± 0.02^bA^	11.37 ± 0.01^bB^
L09	17.67 ± 0.88^cA^	13.33 ± 1.33^cB^	14.54 ± 0.42^bB^	21.36 ± 0.38^bA^	20.85 ± 0.09^cA^	13.59 ± 0.01^cB^	10.97 ± 0.03^cA^	5.11 ± 0.01^cB^
L10	23.33 ± 1.33^bA^	21.33 ± 0.33^aA^	10.88 ± 0.21^cA^	13.74 ± 0.55^cA^	29.11 ± 0.06^aA^	27.91 ± 0.09^aB^	23.69 ± 0.02^aA^	16.51 ± 0.03^aB^
L11	26.33 ± 0.67^aA^	20.67 ± 1.20^aB^	15.92 ± 0.43^aB^	21.16 ± 0.19^bA^	23.61 ± 0.04^bA^	15.34 ± 0.01^bB^	13.27 ± 0.03^bA^	5.91 ± 0.01^cB^
L12	23.67 ± 1.20^bA^	17.67 ± 0.33^bB^	18.38 ± 0.23^aB^	27.95 ± 0.90^aA^	15.27 ± 0.71^dA^	10.79 ± 0.01^cB^	9.46 ± 0.06^cA^	3.93 ± 0.01^ dB^
L13	23.67 ± 0.33^bA^	15.00 ± 1.15^bB^	12.14 ± 0.26^bB^	19.49 ± 0.42^bA^	20.07 ± 0.03^cA^	15.39 ± 0.01^bB^	11.06 ± 0.06^cA^	7.62 ± 0.01^cB^

Means are provided with error bars. Small letters indicate a significant (*p* ≤ 0.05) difference between locations and capital letters between treatments of *Cynodon dactylon*. Abbreviations are given at the end of the manuscript

*Abbreviations*: *NT* net tiller, *EL* electrolyte leakage, *FW* fresh weight, *DW* dry weight

### Chlorophyll contents

In our study, chlorophyll a reduced Chl *b* and carotenoids increased while, no significant effect in Chl *a/b* ratio along both longitude and latitude gradients was found. Chl *a* and *b* contents were higher in L04 and L05 under both normal and shade, but Chl *a* was in L05, whereas L01 maintained Chl *a* under both environments. There was no significant difference in Chl *a/b* ratio, but enhanced carotenoid content was noted under normal and shade; however, L02 showed a maximum Chl *a/b* ratio and carotenoids among all the locations in longitudes (Table 7). Along the latitudinal gradient, Chl *a* reduced significantly, but L10 maintained higher rate and Chl *b* content was maintained by L08 and L10 under both control and shade treatments. The chlorophyll *a/b* ratio was unchanged among all the locations, and carotenoids were higher in L08 under both normal and shady environments (Table 8).

**Table 7:** Photosynthetic pigments of *Cynodon dactylon* under control and shade stress along longitude gradient

Var.	Chl *a *(mg g^-1^ FW)		Chl *b *(mg g^-1^ FW)		Chl *a/b *(mg g^-1^ FW)		*Caro. *(mg g^-1^ FW)	
	Control	Shade	Control	Shade	Control	Shade	Control	Shade
L01	3.91±0.04^bA^	3.22±0.01^bA^	1.10±0.01^cB^	2.01±0.01^bA^	2.34±0.01^aA^	2.26±0.01^aA^	1.86±0.03^bB^	3.36±0.09^bA^
L02	3.05±0.67^bA^	1.89±0.01^cB^	2.39±0.02^bA^	2.26±0.02^bA^	3.13±0.03^aA^	3.56±0.01^aA^	4.06±0.01^aA^	5.06±0.09^aA^
L03	2.21±0.01^cA^	1.54±0.13^cB^	2.54±0.01^bA^	2.83±0.01^bA^	2.73±0.03^aA^	2.24±0.00^aA^	2.93±0.03^bB^	4.66±0.02^aA^
L04	6.24±0.38^aA^	3.34±0.03^bB^	3.87±0.34^aA^	3.74±0.00^aA^	2.53±0.02^aA^	1.98±0.01^aA^	2.03±0.09^bA^	3.28±0.30^bA^
L05	6.24±0.13^aA^	5.40±0.03^aB^	3.26±0.03^aA^	3.44±0.03^aA^	2.66±0.01^aA^	2.16±0.02^aA^	3.67±0.01^aA^	4.69±0.09^aA^
L06	3.65±0.01^bA^	2.10±0.04^bB^	1.37±0.03^cA^	1.38±0.02^cA^	2.63±0.02^aA^	2.12±0.01^aA^	2.30±0.04^bA^	3.02±0.01^bA^
L07	3.28±0.02^bA^	2.47±0.10^bB^	1.27±0.02^cA^	1.33±0.01^cA^	2.43±0.01^aA^	2.01±0.01^aA^	1.81±0.08^bA^	2.60±0.02^bA^

Means are provided with error bars. Small letters indicate a significant (p ≤0.05) difference between locations and capital letters between treatments of *Cynodon dactylon*. Abbreviations are given at the end of the manuscript

*Abbreviations*: *Chl a *chlorophyll *a, Chl b, *Chlorophyll *b, Chl a/b *chlorophyll *a/b *ratio, *Caro *Carotenoids

**Table 8:** Photosynthetic pigments of *Cynodon dactylon* under control and shade stress along latitude gradient

Var	Chl *a* (mg g^−1^ FW)		Chl *b* (mg g^−1^ FW)		Chl *a/b* (mg g^−1^ FW)		*Caro.* (mg g^−1^ FW)	
	Control	Shade	Control	Shade	Control	Shade	Control	Shade
L08	6.32 ± 0.09^aA^	3.03 ± 0.01^bB^	2.30 ± 0.13^aA^	2.53 ± 0.02^aA^	2.65 ± 0.01^aA^	2.36 ± 0.02^aA^	3.56 ± 0.01^aA^	3.95 ± 0.02^aA^
L09	3.72 ± 0.05^bA^	2.27 ± 0.08^bB^	1.38 ± 0.03^aA^	1.63 ± 0.02^bA^	2.65 ± 0.02^aA^	2.04 ± 0.01^aA^	2.47 ± 0.10^bB^	3.10 ± 0.05^aA^
L10	7.11 ± 0.06^aA^	7.03 ± 0.08^aA^	2.66 ± 0.04^bB^	3.17 ± 0.00^aA^	2.66 ± 0.01^aA^	2.59 ± 0.01^aA^	2.44 ± 0.05^bB^	3.45 ± 0.02^aA^
L11	2.41 ± 0.10^bA^	2.06 ± 0.07^bA^	0.96 ± 0.02^bB^	1.26 ± 0.03^bA^	2.58 ± 0.01^aA^	2.23 ± 0.01^aA^	1.29 ± 0.16^bB^	2.07 ± 0.01^aA^
L12	5.90 ± 0.31^aA^	3.93 ± 0.21^bB^	1.07 ± 0.07^aA^	1.63 ± 0.07^bA^	2.36 ± 0.01^aA^	2.02 ± 0.01^aA^	1.04 ± 0.04^aA^	1.99 ± 0.04^aA^
L13	2.12 ± 0.10^bA^	1.70 ± 0.37^cB^	1.84 ± 0.06^aA^	1.95 ± 0.06^bA^	2.43 ± 0.01^aA^	2.11 ± 0.01^aA^	1.17 ± 0.05^bB^	2.71 ± 0.05^aA^

Means are provided with error bars. Small letters indicate a significant (*p* ≤ 0.05) difference between locations and capital letters between treatments of *Cynodon dactylon* locations. Abbreviations are given at the end of the manuscript

*Abbreviations*: *Chl a* chlorophyll *a*, *Chl b* Chlorophyll *b*, *Chl a/b* chlorophyll *a/b* ratio, *Caro* carotenoids

### Photosynthetic traits

**Fig. 1:**
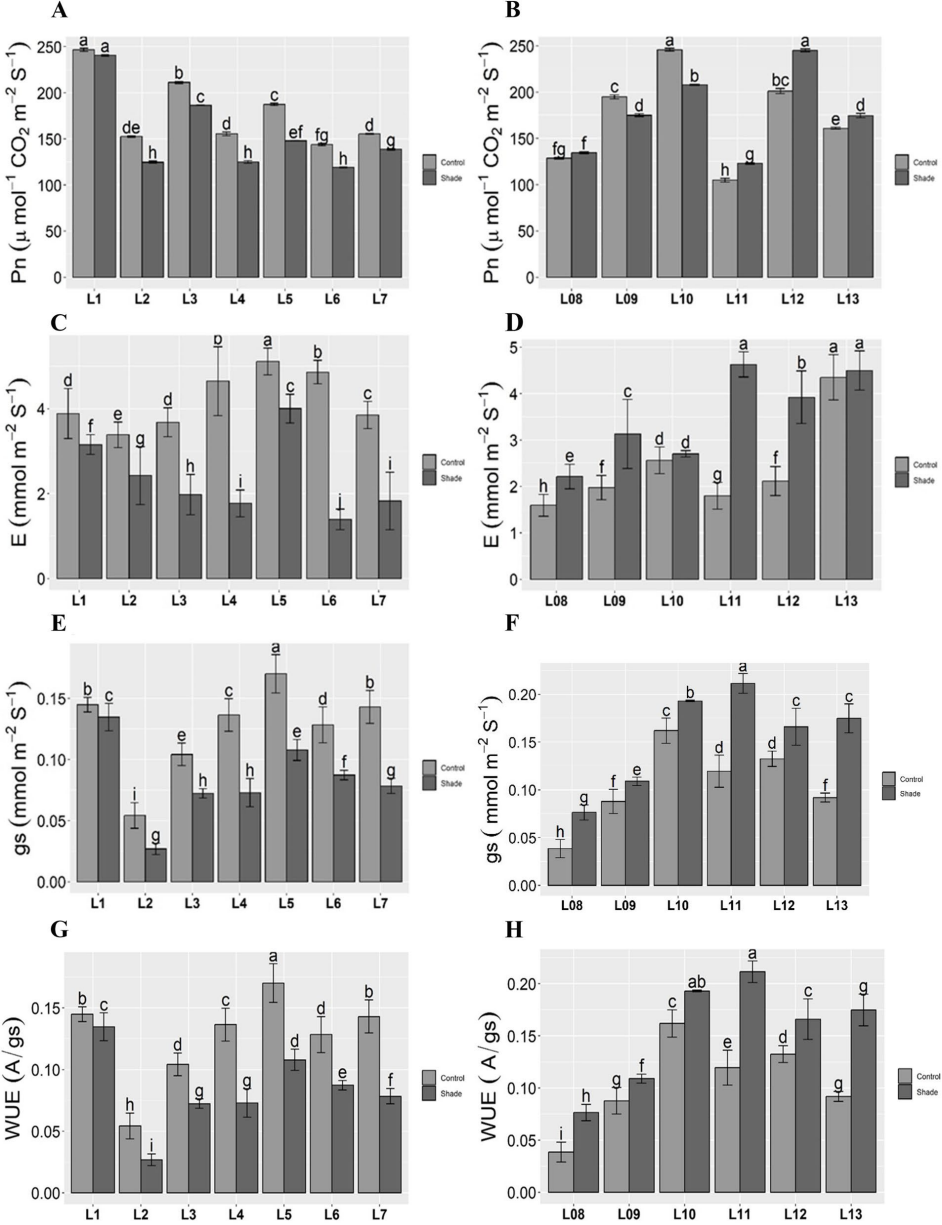
Photosynthetic traits along longitudinal and latitudinal gradient under shade stress. Means ± SE are provided. Bars sharing same lowercase letters are not-significant (*p* ≤ 0.05) in response to control and shade conditions. Along longitude gradient: Abbreviations: **A ***Pn*, The net CO_2_ assimilation rate. **C ***E*, The transpiration rate. **E ***gs*, The stomatal conductance. **G** WUE, The water use efficiency. L1- Tianshui; L2- Baoji; L3- Sanmenxia; L4- Zhengzhou; L-5 Shanxian; L-6 Zaozhuang; L7- Lianyungang. Along latitude gradient: Abbreviations: **B ***Pn*, The net CO_2_ assimilation rate. **D ***E*, The transpiration rate. **F ***gs*, The stomatal conductance; (**H**) WUE, The water use efficiency. L-8 Zhongshan; L-9 Youxian; L-10 Linxiang; L-11 Xiaochang; L-12 Zhumadian; L-13 Cixian

The net CO2 assimilation rate (A) was reduced under shade stress. Still, it was highest in L01 and lowest in L6 under control condition While, under shade stress, L01 showed less reduction in net CO_2_ rate and L2, L4, and L6 showed the lowest rate among all longitudinal locations (Fig. 1A). Along the latitudinal gradient, the maximum rate was observed in L10, and the minimum was observed in L11 under control. Under shade stress, L12 showed the highest but lowest net CO2 rate in L11 (Fig. 1B). The transpiration rate (E) decreased considerably under shaded environment. A higher level of *(E)* was shown by L5 and the lowest by L2 under control. Whereas, Highest in L1 and lowest in L6 under shade along longitude (Fig. 1C) but along a latitudinal gradient, L13 showed the highest and L08 showed the lowest rate under control. More rate was shown by L13 and less by L08 (Fig. 1D). Stomatal conductance (*gs*) was significantly (p ≤ 0.05) increased in L5 and decreased in L2, but less reduction was observed in L1 and more reduction in L3 and L4 under control and shade, respectively, along longitude (Fig. 1E). Along latitude, maximum *gs* rate was observed in L10 and minimum in L11 under control, but under shade, it was maximum in L11 and minimum in L08 (Fig. 1F). Water use efficiency (WUE) decreased significantly (p ≤ 0.05) in both gradients. Maximum rate was observed by L5 and minimum L2 under control whereas maximum in L1 and minimum in L2 under shade stress in longitudinal gradient (Fig. 1G). The highest rate was revealed as L10, the lowest by L08, and the maximum in L11 and minimum in L08 under control and shade, respectively, along latitude (Fig. 1H).

### Organic osmolytes

**Fig. 2:**
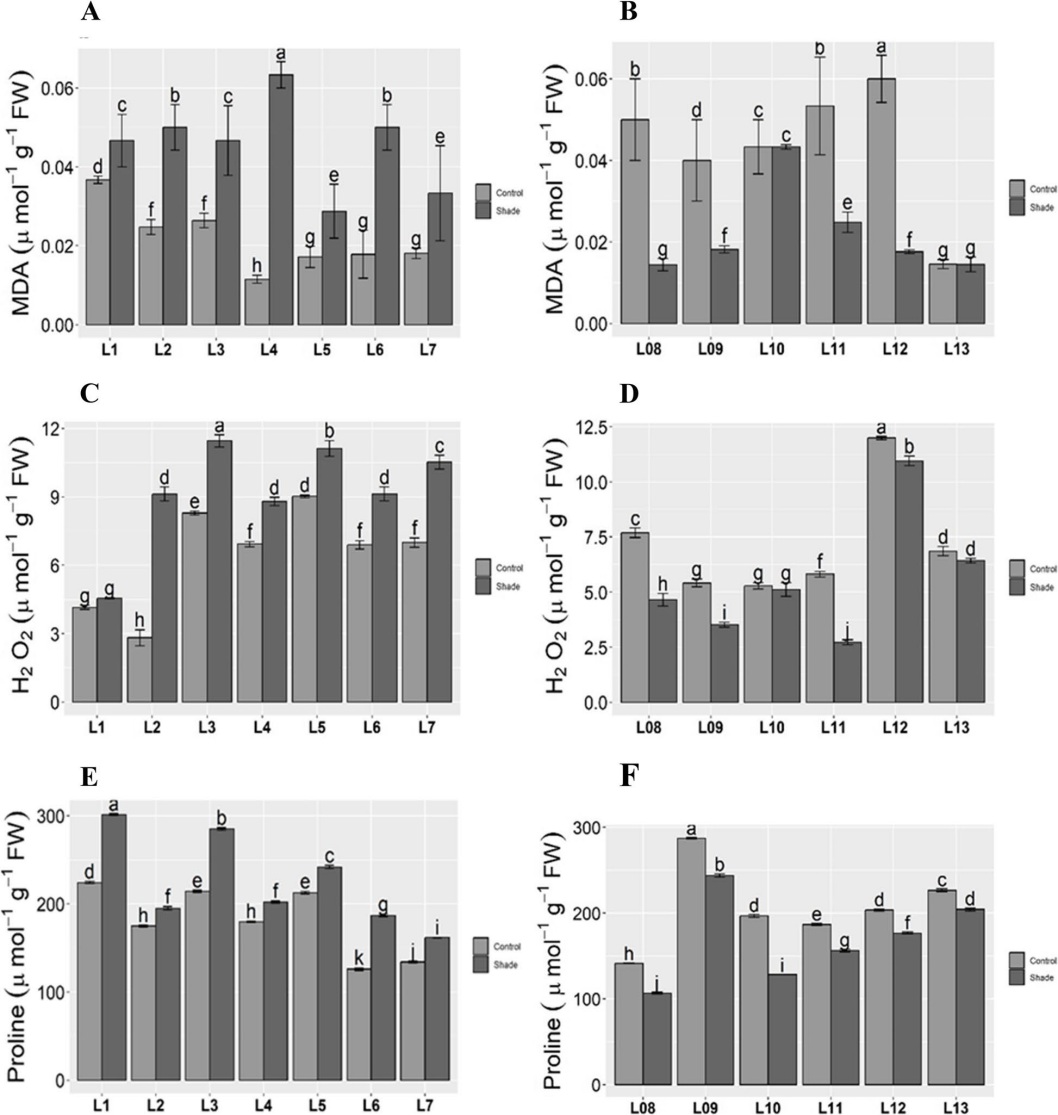
Lipid peroxidation (MDA), ROS (H_2_O_2_) and Organic osmolytes (Proline) of *Bermuda* grass under shade stress collected from different longitudes and latitude gradients. Means ± SE are provided. Bars sharing same lowercase letters are non significant (*p* ≤ 0.05) in response to control and shade conditions. Along longitude: **A** Proline. **C** MDA, malondialdehyde. **E** H_2_O_2_, hydrogen peroxide. Abbreviations: L1- Tianshui; L2- Baoji; L3- Sanmenxia; L4- Zhengzhou; L-5 Shanxian; L-6 Zaozhuang; L7- Lianyungang. Along latitude: **B** Proline. **D** MDA, malondialdehyde. **F** H_2_O_2_, hydrogen peroxide. Abbreviations: L-8 Zhongshan; L-9 Youxian; L-10 Linxiang; L-11 Xiaochang; L-12 Zhumadian; L-13 Cixian

Lipid peroxidation of the membrane was measured as malondialdehyde content. MDA increased significantly under shade in both gradients. Maximum MDA was observed in L01 under control but minimum in L04, while under shade, it was highest in L04 and lowest in L07 along the longitudinal gradient (Fig. 2A). Along the latitudinal gradient, it was highest in L12 and lowest in L13 under normal conditions while L10 showed maximum, but L08 and L13 showed minimum MDA content under low light stress (Fig. 2B). Reactive oxygen species was measured by measuring H_2_O_2_ content. The highest level was observed in L05 and L03 and the minimum by L02 and L01 under normal and the same trend in the shade was depicted in longitudinal regions (Fig. 2C). In latitudinal areas, the maximum quantity was observed by L12 and minimum L09 and L10 in controlled environment, but in low light, it was high in L12 and lowest in L11 (Fig. 2D). In organic osmolytes, proline significantly (p ≤ 0.05) increased under shaded condition The highest proline content was observed in L01 in both control and shade treatment. Whereas the decreased rate in L06 under control and L07 in the shade was observed along longitude (Fig. 2E). However, in latitude, the maximum pro quantity was shown by L09, but L08 showed the minimum under both control and shaded conditions (Fig. 2F).

### Enzymatic antioxidants

**Fig. 3:**
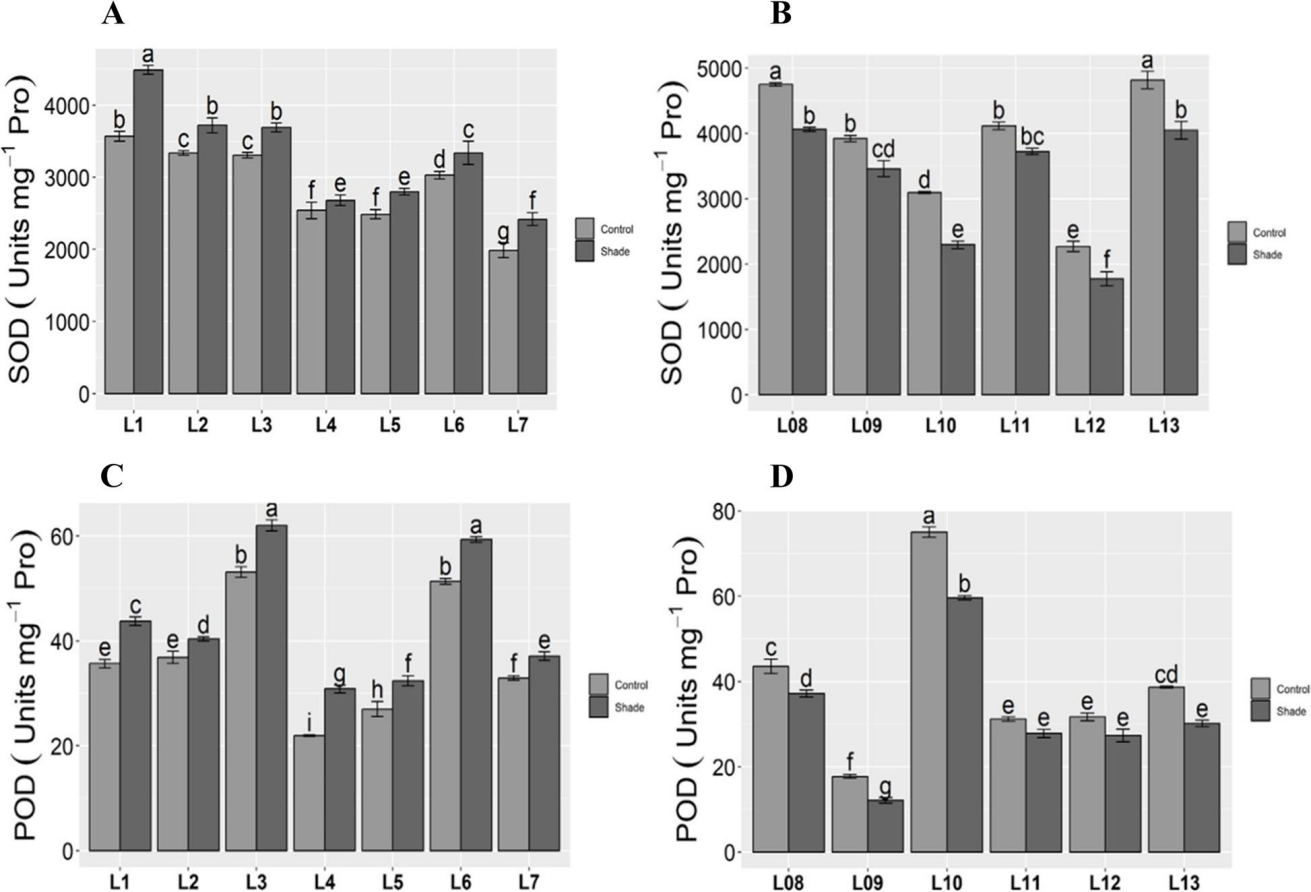
Enzymatic antioxidants in bermudagrass along longitude and latitude gradient. Means ± SE are provided. Bars sharing same lowercase letters are not-significant (*p* ≤ 0.05) in response to control and shaded conditions. Along longitude: **A** SOD, superoxide dismutase. **C** POD, peroxide dismutase. Abbreviations: L1- Tianshui; L2- Baoji; L3- Sanmenxia; L4- Zhengzhou; L-5 Shanxian; L-6 Zaozhuang; L7- Lianyungang. Along latitude: **B** SOD, superoxide dismutase. **D** POD, peroxide dismutase. Abbreviations: L-8 Zhongshan; L-9 Youxian; L-10 Linxiang; L-11, Xiaochang; L-12 Zhumadian; L-13 Cixian

Cellular antioxidants normally increased significantly when bermudagrass was subjected to shade stress. Along longitudes, SOD content was maximum in L01 and minimum in L07 under both control and shady environments (Fig. 3A). The L08 and L13 showed the highest under control and shade L12 showed the lowest SOD rate along latitudes (Fig. 3B). The POD accumulation was higher in L03 and L06 and smaller in L04 under both control and shade (Fig. 3C). Along the latitudinal gradient L09 and L10 accumulated a higher quantity of POD in both normal and low-light conditions (Fig. 3D).

### Gene expression under shade stress

**Fig. 4:**
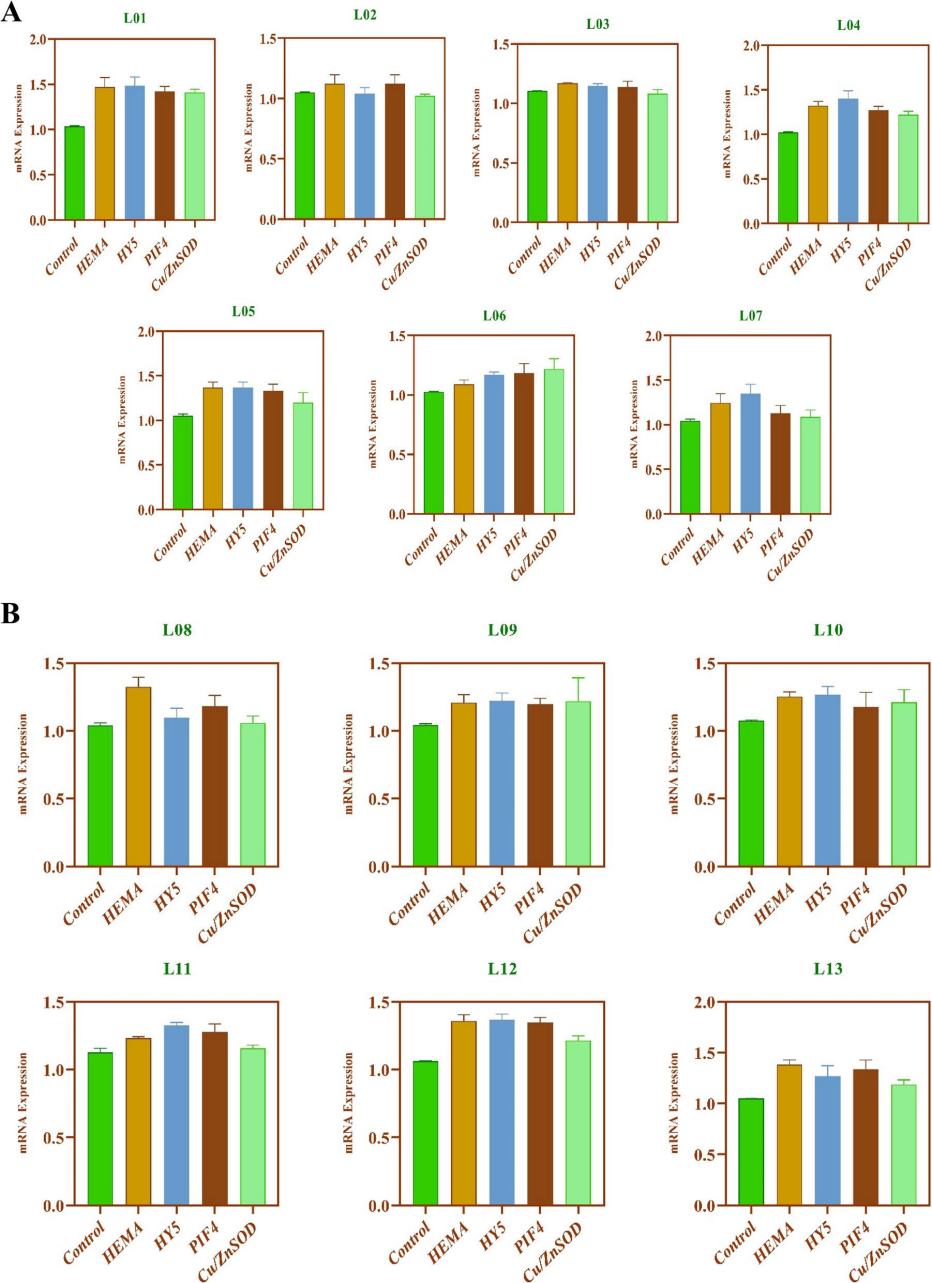
**A** Gene expression in response to shaded conditions in Bermudagrass from different longitudes. **B** Gene expression in response to shaded conditions in Bermudagrass from different latitudes

The expression level of four genes was studied, and three were related to photosynthetic capacity, photosynthetic pigments production, and phytochrome interacting factors. One was an enzymatic antioxidant-related gene. All genes showed a differential expression level in different localities along a longitudinal gradient. *HEMA* (glutamyl-tRNA reductase) was up-regulated in all the localities, but the highest expression was observed in L01. *HY5* expression level was highest in L01, L04, and L07. As with increasing the longitude, gene expression level was also increased. The *PIF4* gene was highly expressed in L01, L04, and L06 locations without the effect of the region. *Cu/ZnSOD* overly-expressed in L01 but expression level decreased in the genotypes of higher longitudes The L07 showed no expression of *Cu/ZnSOD* under shade stress (Fig. 4A). Different genes showed different expression levels in shaded environments along the latitudinal gradient. *HEMA* gene up-regulated in all the localities, but high expression was observed in L08, L10, and L12. *HY5* was overly expressed in L10, L11 and L12, and *PIF4* showed highest expression in L09 and L12. *Cu/ZnSOD* was expressed in all localities except L08 and L11, and there is a higher expression in L09 and L10 (Fig. 4B). All the examined genes up-regulated, by enhancing the photosynthetic activity by producing sound chlorophyll content, showing that bermudagrass germplasm used in the current study are shade-tolerant.

### Principal component analysis

**Fig. 5:**
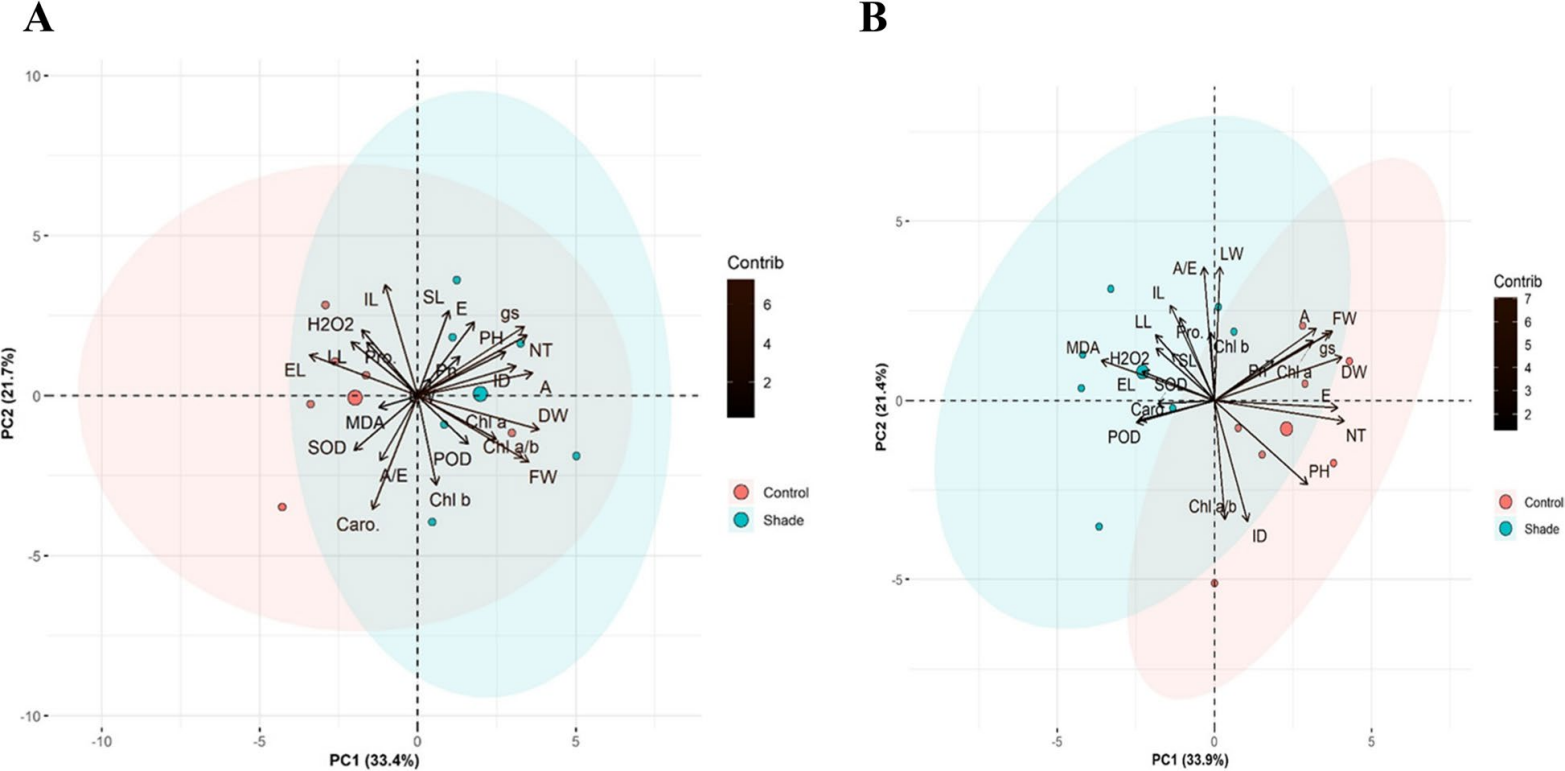
PCA biplot of morphology, photosynthetic efficiency, pigments, organic osmolytes and cellular antioxidants of *Cynodon dactylon* under controlled and shade treatments. Abbreviations: C, Control; S, Shade; L, Locations; PH, plant height; SL, stolon length; IL, internode length; ID, internode diameter; LL, leaf length; LW, leaf width; FW, fresh weight; DW, dry weight; EL, electrolyte leakage; NT, net tiller; Pn, net CO_2_ rate; gs, stomatal conductance; E, transpiration rate; A/E, water use efficiency; H_2_O_2_, hydrogen peroxide; MDA, malondialdehyde; Chl a, chlorophyll a content; Chl b, Chlorophyll b content; Chl a/b, chlorophyll a/b ratio; Caro, carotenoids; Pro, proline; SOD, superoxide dismutase; POD, peroxide dismutase

The PCAs for *Cynodon dactylon* showed a significant (p ≤ 0.05) variation as PC1 33.9% and PC2 21.4% (total; 55.3%) under control and shade treatments at various longitude gradients (Fig. 5A). In the control group, L04 was tightly linked with A, dry weight was closer to L05, and Chl *a* was closely grouped with L01 by showing loading toward PC1 with higher positive eigenvalues. While under shade, L06 was closely associated with control L03, and PH correlated with control L07, and ID with Chl a/b with negative eigenvalues. In the shade ellipsed, Pro was close to SL and SOD, MDA and H_2_O_2_ were associated with each other concerning shade L01 and L04 and plotted toward the PC2 exhibiting the positive eigenvalues. However, Caro was related to POD with lower negative eigenvalues.

The PCAs for latitudinal gradient showed a significant (p ≤ 0.05) variation as PC1 33.4% and PC2 21.7% (total; 55.1%) under control and shade treatments (Fig. 5B). In shade, SL was closely associated with E, gs with PH and NT, SL with SL12 with positive eigenvalues (± 2). The negative eigenvalues were drawn by control L11: DW, Chl: Cl8, Chl a/b: FW, and POD: shade L09. In control, H_2_O_2_ was associated with control L12, Pro, and LL. The MDA was closely linked with SOD, while Caro is somehow related to A/E with negative eigenvalues.

### Correlation matrix and clustered heatmaps

**Fig. 6:**
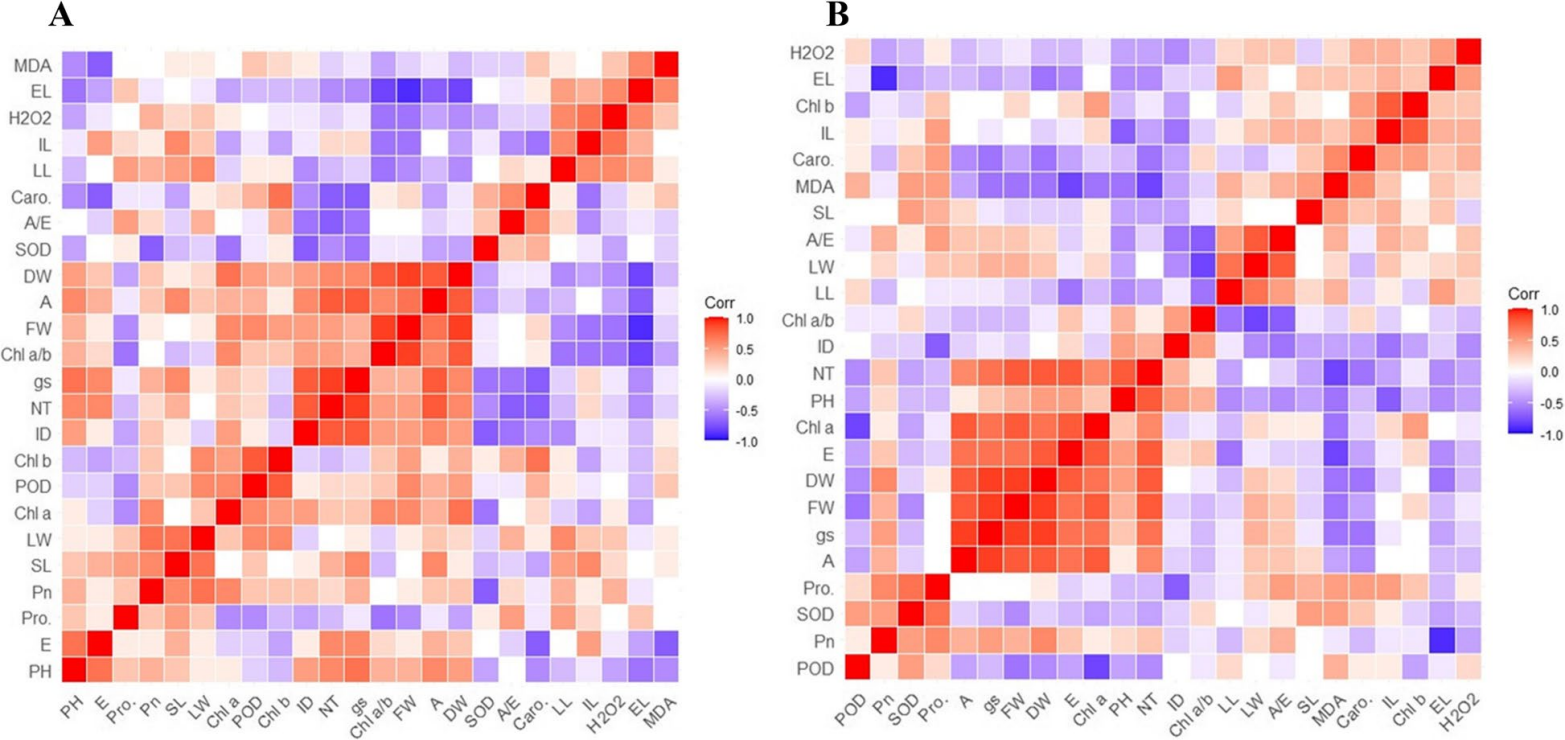
Correlation matrix of morphology, physiology and photosynthetic pigments of *Cynodon dactylon* in response to control and shaded conditions. **A** along longitude **B** along latitude gradient. Abbreviations: C, Control; S, Shade; L, Locations; PH, plant height; SL, stolon length; IL, internode length; ID, internode diameter; LL, leaf length; LW, leaf width; FW, fresh weight; DW, dry weight; EL, electrolyte leakage; NT, net tiller; Pn, net CO_2_ rate; gs, stomatal conductance; E, transpiration rate; A/E, water use efficiency; H_2_O_2_, hydrogen peroxide; MDA, malondialdehyde; Chl a, chlorophyll a content; Chl b, Chlorophyll b content; Chl a/b, chlorophyll a/b ratio; Caro, carotenoids; Pro, proline; SOD, superoxide dismutase; POD, peroxide dismutase

For longitude, the H_2_O_2_ was positively correlated with Pro, LL, LW, and MDA contents while showing a negative association with A, gs, FW, DW, and E. The EL was positively correlated with LL, LW, SL, Caro, Chl b, and EL while exhibiting a negative correlation with SOD, POD and MDA contents. The MDA contents were shown a significant positive correlation with SOD, POD, and Pro, while a negative association with photosynthetic pigments and morphological traits of *C. dactylon*. Also, the antioxidant SOD and POD contributed to growth traits (Fig. 6A). In response to latitudinal gradients, MDA contents were negatively influenced by SL, LW, Chl a, LL, and A/E. The H_2_O_2_ also showed a negative correlation with photosynthetic pigments and fresh and dry weights (FW, DW). The LL was positively correlated with Pn, SL, LW, and Chl a. The Caro was significantly and positively associated with Chl b and ID (Fig. 6B).

**Fig. 7:**
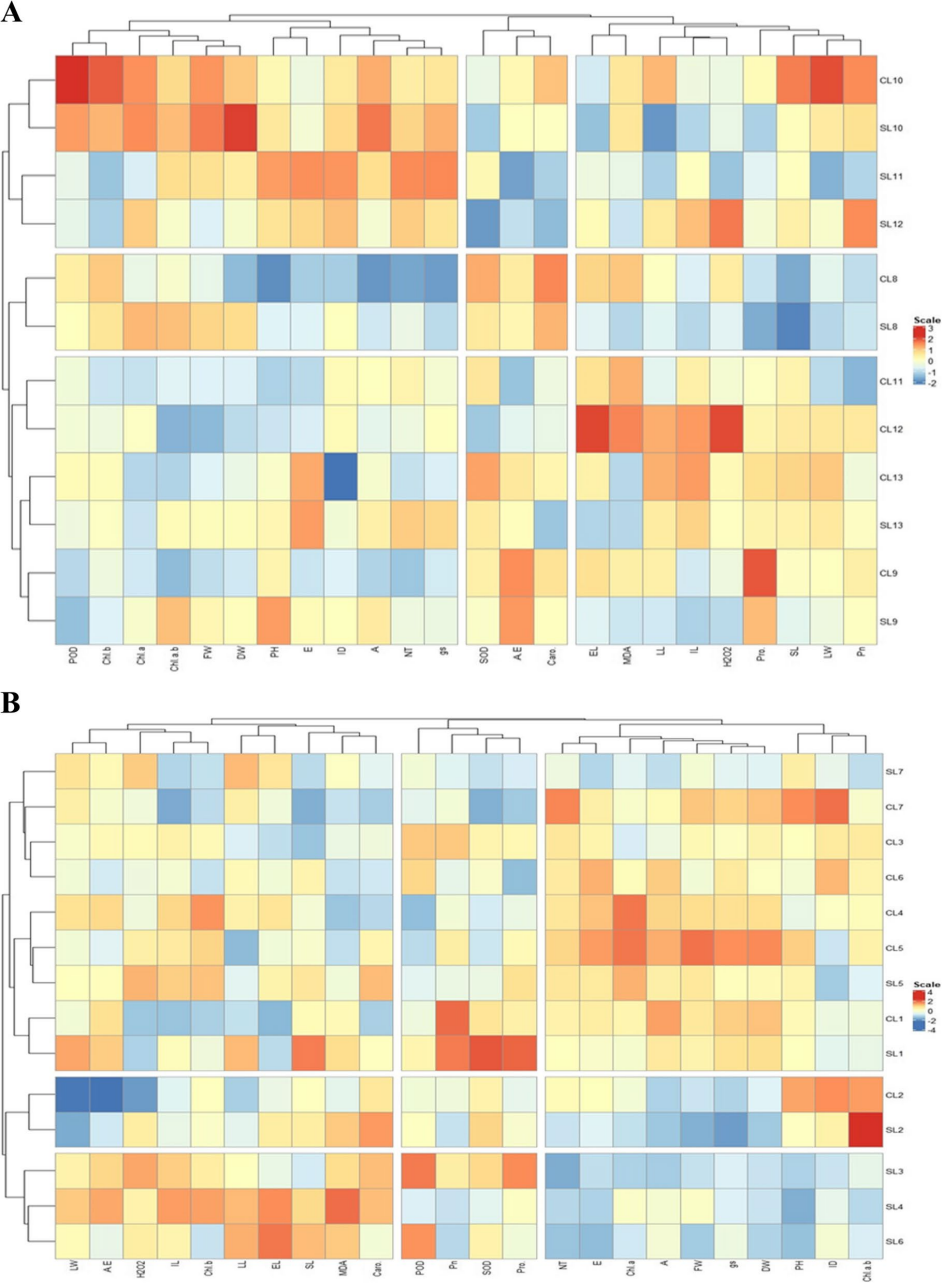
Clustered heatmap of morpho-physiological and photosynthetic traits of *Cynodon dactylon* in response to controlled and shade treatments **(A)** along longitude **(B)** along latitude gradient. Abbreviations: C, Control; S, Shade; L, Locations; PH, plant height; SL, stolon length; IL, internode length; ID, internode diameter; LL, leaf length; LW, leaf width; FW, fresh weight; DW, dry weight; EL, electrolyte leakage; NT, net tiller; Pn, net CO_2_ rate; gs, stomatal conductance; E, transpiration rate; A/E, water use efficiency; H_2_O_2_, hydrogen peroxide; MDA, malondialdehyde; Chl a, chlorophyll a content; Chl b, Chlorophyll b content; Chl a/b, chlorophyll a/b ratio; Caro, carotenoids; Pro, proline; SOD, superoxide dismutase; POD, peroxide dismutase

A clustered heatmap was constructed to evaluate the response of *C. dactylon* under shade and control. In response to longitude, the Chl a/b was linked with shade L02 and control L02, while ID was related to control L6, L07, and L2. The gs and DW were strongly associated with control L05 and L01, while Chl a and E were linked with control L04 and L05. The Pn, SOD, POD and Pro were tightly and positively linked with control L01 and shade l1. The LL, EL, SL, MDA, and Caro were significantly influenced under shade L06 and L04 (Fig. 7A). Under latitude gradients, the POD, Chl b, Chl a, Chl a/b, FW, DW, PH, and GS were tightly linked with control L10, shade L10, and shade L11. However, these traits have negatively influenced control L08 and shade L08 (Fig. 7B).

## Discussions

Normally, management of turfgrass under shaded conditions varies at cell, tissue, organ andwhole-plant level. Turfgrass's response to shade depends upon growth percentage and recovery from stress. As an extensively distributed grass, bermudagrass of same cultivars may differ in shade tolerance because of different primary environments. The selection of improved genotypes to reduce the negative impacts of shade are recommended for low-light environments rather than other management strategies [10].

Primarily, the plant modifications to shaded conditions are reflected in its appearance [14]. These adaptations may increase the survival of plants under low light or shading environments. Different climatic conditions may influence the plant phenotypic characteristics between latitudinal and longitudinal gradients, reflecting phenotypic plasticity and adaptive evolution [56].

In our study, there was reduction in morphological traits, mainly plant height, internode diameter, leaf width, and the number of tillers. While stolon length, internode length and leaf length was enhanced under shaded conditions. The reduction in photosynthetic rate and biomass during plant development ultimately resulted in reduced total fresh and dry weight of the plant. Our findings are similar to Shi et al., [41] in that under shade stress, photosynthetic efficiency reduces. In shade-tolerant bermudagrass, more vertical growth captures more light, resulting in increased stolon, internode, and leaf length. Whereas leaves with more length and area but with reduced width considered to survive under shade. Our current results are in accordance with Cao et al., [4].

The balance between water uptake and transpiration rate measures a plant's water status or osmotic balance. Plants respond to shade by accumulating osmotic solutes to maintain cell turgor, cell development, and metabolism. Soluble protein, sugar, and free proline contents increased in response to the shade stress [39]. Proline is a type of amino acid that makes-up plant proteins. Normally, it exists freely in plants. However, when plants experience various environmental stresses, proline accumulates in cell cytoplasm in large quantities to regulate osmotic potential. This helps to maintain normal growth under stressed environment [30], and these results are in accordance with Vasilakoglou et al., [49]. While electrolyte leakage is the imbalance of ions released from leaf tissue or plasma membrane because of membrane injury under stress, similar to Zhu [57].

The photosynthetic rate and pigments can illustrate the physiological performance of grasses. Low light intensities decrease the photosynthetic activity and transpiration rate. Warm-season turfgrasses are most active in photosynthesis at temperatures between 27–35 °C. In addition, a difference in stomatal conductance was also observed. It is clear in shaded environments with excessive moisture in the root area, stomatal conductance and transpiration rate increase, which helps the grasses avoid waterlogging. Under shaded conditions, internal carbon dioxide concentrations and chlorophyll contents decreased significantly. The fluctuations in available light can influence the chlorophyll contents under both sunny and shady environments, in align with the findings of Malik et al., [26].

It is indisputable that warm-season grasses do not perform optimally in limited light conditions. Specifically, bermudagrass experiences a significant reduction in chlorophyll *a* (Chl *a*) content and no significant effect on Chl *a/b* ratio, while the total chlorophyll and Chl b contents increased in our study. This is primarily due to the acceleration in chlorophyll a conversion to chlorophyll b in bermudagrass leaves in shaded environments, which results in a high increase rate in chlorophyll b as compared to chlorophyll a, subsequently decreases the ratio of chlorophyll a/b [51]. On the other hand, *HEMA* (glutamyl-tRNA reductase) is an indispensable gene that plays a key role in initiating chlorophyll production. It is a significant regulator of metabolic and environmental processes that enhances photosynthesis and is up-regulated in plants under low light conditions [38].

Optimum light is obligatory for photosynthesis, producing carbohydrates for plant growth and respiration. Photosynthesis exclusively happens during daylight hours above freezing temperatures, and its efficiency is low until the temperature reaches about 4 °C. Respiration increases with temperature, and if it outpaces photosynthesis, the turf dies [45].

In shaded conditions, plants inevitably face limited light quantum, leading to decreased in leaf temperature, stomatal limitation, stomatal conductance, intercellular CO_2_ concentration, and ultimately photosynthetic rate [52]. Changes in the red/far-red (R/FR) ratios decrease plant photosynthesis. The photosensitive pigment system responds to external environmental light stimulation and has two crucial forms of photosensitive pigments: Pfr (active form) and Pr (passive form). As a result, light can transform the two different forms of photosensitive pigments in plants [5]. Phytochromes are a type of photoreceptor found in plants that act as red/far red-light sensors. They exist in two forms: Pr and Pfr. When exposed to red light, PHYs are activated as the Pr form is converted to the Pfr form.Conversely, far redlight inactivates Pfr by converting it back to the Pr form. This light signal transduction mechanism is essential for plants to perceive and respond to their surrounding environment [21]. Several kinds of phytochromes present in plants, such as PhyA, PhyB, PhyC, PhyD, and PhyE [33], and their homologous genes, such as *HY5* was up-regulated in bermudagrass under low light stress, similar to the study of Liu et al., [23]. PIFs (Phytochrome-interacting factors) have been identified as phytochromes' primary signalling partners. The *PIF4* gene expression level has been extensively studied in bermudagrass. It is undoubtedly established that it regulates multiple plant functions including stomatal development, chlorophyll deprivation, leaf senescence in dark, tolerance against freezing, hypocotyl elongation, and early flowering response to high temperature [37]. Moreover, it effectively regulates hypocotyl elongation and early flowering against light, diurnal conditions, and high temperatures. In accordance with Lorrain et al., [24] as it regulated stomatal movement and chlorophyll content in tolerant varieties. In sensitive genotypes, the down-regulation of *PIF4* resulted in deprevation of chlorophyll contents.

Reactive oxygen species (ROS) are cellular byproducts that perform vital functions such as cell signalling, differentiation, and death. However, under abiotic stress, plants constantly produce ROS during growth, and its production exceeds normal levels, leading to oxidative stress and, inevitably, cell death. The three main ROS types are H_2_O_2,_ OH and ^1^O_2_. Excessive production of ROS oxidizes membrane lipids, proteins, and enzymes, leading to chloroplast and cell dysfunction. High light stress increases membrane permeability, MDA, and carbonyl content, causing oxidative damage. Lipid peroxidation is a common response to low light stress, reflecting the plant's stress level. Low light also profoundly affects plant metabolism and membrane protection. Protective antioxidant enzymes SOD and POD activity notably increased to scavenge excessive ROS, while CAT decreases under low light conditions, as in Xu et al., [53] (Xu, Sun et al. 2010). Moreover, an increase in the activity of the enzymes during abiotic stresses is a dire need for plants to counteract ROS such as H_2_O_2_, free radicals, and signlet oxygen to prevent stress-induced oxidative damage. In the following, the antioxidants enzyme activitywere significantly enhanced to scavenge the excessive lipid peroxidation, H_2_O_2_ and stabilize the cellular membranes.

The metalloenzyme SOD (superoxide dismutase) usually converts O_2_ − • to O_2_ and H_2_O_2_. On the base of metal ion in its active site, it is categorized as copper and zinc (Cu/Zn SOD), manganese (MnSOD) or iron (FeSOD) comprising SODs [29]. *Cu/ZnSOD* exists in the cytosol and chloroplast of plant cells, and MnSOD is present in mitochondrial matrix and peroxisomes. It is confirmed that its relevant gene's expression controls an antioxidant enzyme's activity. The *Cu/ZnSOD* gene upregulation is associated with combating oxidative damage caused by abiotic stress and showed a crucial role in plant survival mechanisms in tolerant genotypes of our study. In accordance with Hu et al., [17] that, *Cu/ZnSOD* was upregulated in tall fescue, tobacco, potato, rice, and brassica.

## Conclusions and future perspectives

The effect of 50% shade was assessed in different *Cynodon dactylon* germplasm along longitudinal and latitudinal gradients of China. The different germplasm responded differentially under shade conditions by securing better morphological traits in terms of PH, SL, ID, LW, growth parameters as NT, FW, DW, lower EL and higher organic osmolytes like proline. The photosynthetic performance was efficient regarding Pn, less transpiration rate reduction and more stomatal conductance. The tolerant cultivars showed higher WUE, lower ROS and MDA contents. The more accumulation of photosynthetic pigments (Chl *a*, *b* and *a* + *b*) and up-regulation of relevant genes (*HEMA*, *HY5*, *PIF4* and *Cu/ZnSOD*) assessed. The present work results demonstrated that various germplasm of the *Cynodon dactylon* has the potential to mitigate shade and can easily grow in low light extremes. The current study provides a broad-scale evaluation of longitudinal and latitudinal differences in traits and new insight into biotic and abiotic factors. The newly introduced species can be excellent germplasm for future studies on shade tolerance mechanisms and pathways involved in it. The genes with up-regulated expression can be transformed into crops to meet the food scarcity. Thus, common bermudagrass can be suggested in areas with limited sunlight. Further research is needed to explore its molecular level characteristics.

## Data Availability

Data will be available on reasonable request from the corresponding author.

## Abbreviations

C: Control
S: Shade
L: Locations
PH: Plant height
SL: Stolon length
IL: Internode length
ID: Internode diameter
LL: Leaf length
LW: Leaf width
FW: Fresh weight
DW: Dry weight
EL: Electrolyte leakage
NT: Net tiller
*Pn*: The net CO_2_ assimilation rate
*E*: The transpiration rate
*Gs*: The stomatal conductance
WUE: The water use efficiency
MDA: Malondialdehyde
H_2_O_2_: Hydrogen peroxide
SOD: Superoxide dismutase
POD: Peroxidase
